# Clinical significance of smear positivity for acid-fast bacilli after ≥5 months of treatment in patients with drug-susceptible pulmonary tuberculosis

**DOI:** 10.1097/MD.0000000000004540

**Published:** 2016-08-07

**Authors:** Hyung Koo Kang, Byeong-Ho Jeong, Hyun Lee, Hye Yun Park, Kyeongman Jeon, Hee Jae Huh, Chang-Seok Ki, Nam Yong Lee, Won-Jung Koh

**Affiliations:** a Department of Internal Medicine, Division of Pulmonary and Critical Care Medicine, Ilsan Paik Hospital, Inje University School of Medicine, Goyang, Gyeonggi; b Department of Medicine, Division of Pulmonary and Critical Care Medicine; c Department of Laboratory Medicine and Genetics, Samsung Medical Center, Sungkyunkwan University School of Medicine, Seoul, South Korea.

**Keywords:** acid-fast bacilli, nontuberculous mycobacteria, Pulmonary tuberculosis, sputum microbiology, treatment outcome

## Abstract

Patients with pulmonary tuberculosis (TB) with acid-fast bacilli (AFB)-positive sputum smear at 5 months or later during treatment are considered to be cases of treatment failure according to World Health Organization guidelines. This study evaluated the proportion, clinical characteristics, and significance of positive sputum smears after ≥5 months of standard treatment in patients with drug-susceptible pulmonary TB.

This was a retrospective cohort study of 1611 patients with culture-confirmed drug-susceptible pulmonary TB who received standard anti-TB treatment from January 2009 to February 2014. Forty-one patients (2.5%) who were smear-positive after ≥5 months of treatment and 123 age- and sex-matched control patients were evaluated.

Among the 41 smear-positive patients, culture of the sputum specimens yielded *Mycobacterium tuberculosis* (MTB) in 1 patient (2.4%), nontuberculous mycobacteria (NTM) in 7 (17.1%), and no growth in the remaining 33 patients (80.5%). Treatment was successfully completed in 40 patients (97.6%) with prolongation of the continuation phase regimens without change to second-line anti-TB treatment. In patients with smear positivity after ≥5 months of treatment compared with controls, cavitation on chest radiographs (53.7% vs. 25.2%, *P* = 0.001), bilateral involvement (51.2% vs. 30.1%, *P* = 0.01) and combined pleural effusion (26.8% vs. 10.6%, *P* = 0.01) were found more frequently at the time of treatment initiation, and paradoxical response occurred more commonly (19.5% vs. 3.3%, *P* = 0.002) during treatment.

Smear-positive sputum after ≥5 months of standard anti-TB treatment was mainly because of nonviable MTB bacilli or NTM in patients with drug-susceptible pulmonary TB. AFB smear alone should not be used to assess treatment failure and careful examination of microbiologic status, including culture and drug susceptibility testing, is needed before making changes to retreatment regimens or empirical second-line anti-TB regimens in these patients.

## 1 Introduction

Tuberculosis (TB) is a major global health concern. In 2014, there were 9.6 million new cases of active TB and 1.5 million deaths worldwide.^[1]^ The standard treatment for TB comprises an intensive phase with isoniazid, rifampicin, pyrazinamide, and ethambutol for 2 months, followed by a continuation phase that comprises the concomitant use of isoniazid and rifampicin for another 4 months.^[2–4]^ This standard treatment is highly effective for drug-susceptible TB.^[2–4]^

Sputum smear microscopy for acid-fast bacilli (AFB) is a widely available, simple, and inexpensive tool for pulmonary TB diagnosis and treatment monitoring.^[5,6]^ Response to TB treatment should be monitored by follow-up sputum smear microscopy.^[7,8]^ Diminishing numbers of AFB to smear-negative status during treatment are considered an indication of treatment success, whereas increasing numbers of AFB to smear-positive status in the later phase of treatment indicate treatment failure.^[9]^ According to World Health Organization (WHO) guidelines, a positive sputum smear in patients with pulmonary TB after ≥5 months of treatment is defined as treatment failure.^[10,11]^ Patients whose previous course of therapy has failed should undergo a retreatment regimen or empirical second-line anti-TB regimen.^[10]^

Because AFB-positive smears may be because of the presence of nonviable *Mycobacterium tuberculosis* (MTB) bacilli or nontuberculous mycobacteria (NTM)^[12,13]^; however, misclassification as treatment failure and unnecessary treatment modifications in these patients may occur in clinical practice and programmatic conditions, especially in resource-limited settings.^[14,15]^ There are limited data in the literature regarding the clinical significance of AFB-positive smears after ≥5 months of standard anti-TB treatment in patients with drug-susceptible pulmonary TB, and previous studies included drug-resistant TB patients or did not have drug susceptibility results fully available.^[16–20]^ The purposes of the present study were to evaluate the prevalence, clinical characteristics, and significance of positive sputum smears after ≥5 months of standard anti-TB treatment in patients with drug-susceptible pulmonary TB.

## 2 Methods

### 2.1 Study subjects

This single-center case–control study was conducted by retrospective medical record review of 2014 patients with culture-confirmed pulmonary TB at Samsung Medical Center (a 1961-bed referral hospital in Seoul, Korea) between January 2009 and February 2014.

**Figure 1:**
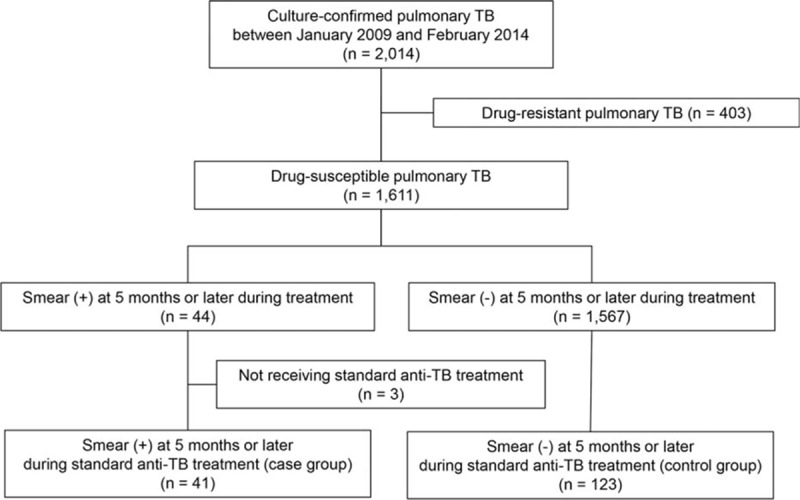
Study population. TB = tuberculosis.

After excluding 403 patients with pulmonary TB in whom MTB isolates were resistant to any first-line TB drug such as isoniazid, rifampicin, ethambutol, and pyrazinamide, 1611 patients with drug-susceptible pulmonary TB were classified into 2 groups according to AFB smear results after ≥5 months of treatment. Among 44 patients with smear-positive sputum at this time, 3 patients who did not receive standard anti-TB treatment because of drug interactions or adverse reactions were excluded. Accordingly, the case group comprised 41 patients with positive smears after ≥5 months of treatment. Using a nested case–control design with age- and sex-matching, we selected 123 patients with negative smear results after ≥5 months of treatment presenting during the same period to be the control group (Fig. 1).

Patient medical records were reviewed to obtain data on medical conditions, symptoms, radiologic findings, anti-TB treatment regimens, treatment outcomes, and serial results of AFB stains and sputum sample cultures. All 164 patients underwent chest radiographs and 157 (95.7%) had available chest-computed tomography data at the time of pulmonary TB diagnosis.

The Institutional Review Board of Samsung Medical Center approved this study, including the review and publishing of information obtained from patient records (IRB no. 2015-09-045). The requirement for informed consent was waived for the use of patient medical data, as all patient information was anonymized and de-identified before analysis.

### 2.2 Diagnostic methods

The AFB smears were examined using an auramine-rhodamine fluorescent stain, followed by confirmation with Ziehl-Neelsen staining. The staining results were graded according to the American Thoracic Society/Centers for Disease Control and Prevention guidelines, and those graded more than 1+ (1–9 bacilli in 100 fields) were defined as smear-positive.^[21,22]^ Cultures were performed using both 3% Ogawa solid medium (Shinyang, Seoul, Korea) and liquid broth medium (mycobacterial growth indicator tube [MGIT]; Becton-Dickinson and Co., Sparks, MD). Positive cultures for MTB were confirmed by MPT64 antigen testing (SD BIOLINE TB Ag MPT64 Rapid; Standard Diagnostics Inc., Yongin-si, South Korea). If any of these tests yielded a negative result, an *rpoB*-specific PCR test using the MTB-ID V3 kit (YD Diagnostics, Yongin-si, South Korea) was performed to differentiate between MTB and NTM. All first isolates of MTB per patient were tested for resistance to isoniazid and rifampin using the MGIT 960 system and were referred to the Korean Institute of Tuberculosis, a WHO-designated Supranational Reference Laboratory, for drug susceptibility testing for all first-line anti-TB drugs.^[23–25]^

### 2.3 Treatment and outcomes

The standard anti-TB regimen is 2 months of daily isoniazid, rifampin, ethambutol, and pyrazinamide (HREZ), and then 4 months of daily isoniazid and rifampin with or without ethambutol (HR[E]). If pyrazinamide cannot be included in the initial phase of treatment, 9 months of daily isoniazid and rifampin with or without ethambutol was administered.^[26]^ Although these regimens were interrupted based on the presence of adverse reactions, all patients restarted standard anti-TB medication within 4 weeks and received anti-TB medication for at least 24 weeks, not including the interrupted periods.

Treatment failure was defined as positive sputum culture of MTB after ≥5 months of treatment in this study.^[11]^ Paradoxical response was defined as a worsening of existing lesions or presentation of new lesions during anti-TB treatment.^[27]^

### 2.4 Statistical analysis

The data are presented as median and interquartile range (IQR) for continuous variables and as numbers (percentage) for categorical variables. The data were compared using the Mann–Whitney *U* test for continuous variables and Pearson *χ*^2^ test or Fisher exact test for categorical variables. Multivariate logistic regression analysis was used to determine independent variables that predicted positive AFB smear after ≥5 months of treatment. Five objective variables with *P* < 0.1 in univariable analysis were included for multivariate logistic regression analysis. All tests were 2-sided and a *P* value of <0.05 was considered significant. The data were analyzed using PASW Statistics 20 (SPSS Inc, Chicago, IL).

## 3 Results

### 3.1 Baseline characteristics

Among 1611 patients with drug-susceptible pulmonary TB, 41 patients (2.5%) were smear-positive after ≥5 months of standard anti-TB treatment. Of these patients, 28 (68.3%) were males and the median age was 54 years (IQR, 41–68 years). The 123 patients in the control group had the same age and sex distributions as those in the case group (Table 1). None of the patients were infected with human immunodeficiency virus. When the case group was compared with the control group, cavitation (53.7% vs. 25.2%, *P* = 0.001), bilateral involvement (51.2% vs. 30.1%, *P* = 0.01), and combined pleural effusion (26.8% vs. 10.6%, *P* = 0.01) on chest radiography were more frequent, and cough (63.4% vs. 40.7%, *P* = 0.01) and dyspnea (24.4% vs. 10.6%, *P* = 0.03) were more frequent complaints at the time of treatment initiation.

**Table 1:**
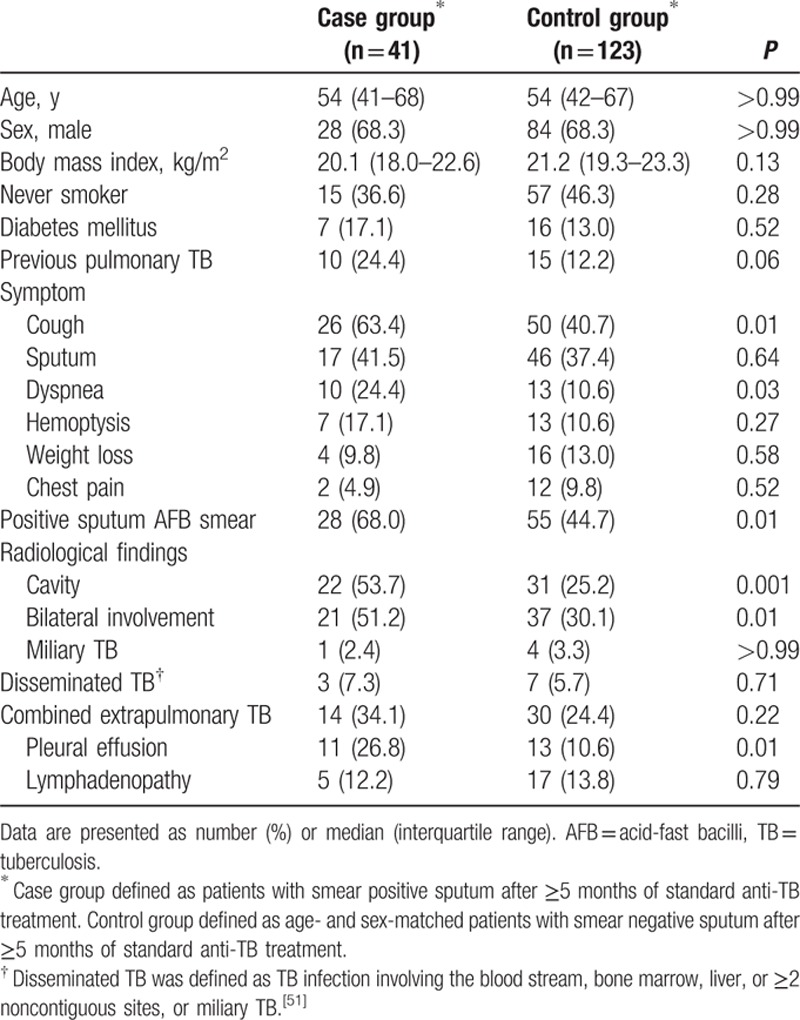
Baseline characteristics according to sputum smear positivity after ≥5 months of anti-TB treatment.

At the time of diagnosis, the proportion of patients with a positive sputum AFB smear was higher in the case group than the control group (68.3% [28/41] vs. 44.7% [55/123], *P* = 0.01). After 2 months of anti-TB treatment, the proportion of patients with a positive sputum AFB smear was also higher in the case group than the control group (56.1% [23/41] vs. 7.3% [9/123], *P* < 0.001).

### 3.2 Culture results of smear-positive sputum specimens after ≥5 months of treatment

**Figure 2:**
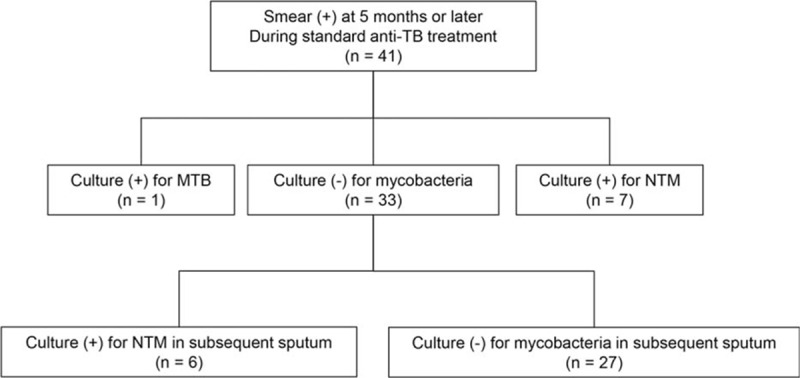
Culture results of smear-positive sputum after ≥5 months of treatment. MTB = *Mycobacterium tuberculosis*, NTM = nontuberculous mycobacteria, TB = tuberculosis.

Of 41 patients with positive smears after ≥5 months of standard anti-TB treatment, culture of the sputum specimens yielded MTB in 1 patient (2.4%), NTM in 7 patients (17.1%), and no growth in the remaining 33 patients (80.5%). Among these 33 patients with sputum labeled no growth, 6 patients (18.2%) were culture-positive for NTM on subsequent sputum specimens (Fig. 2).

### 3.3 Treatment and outcomes

Standard anti-TB medication was initiated in all patients (Table 2). There was no difference in the proportion of patients who received pyrazinamide between the case and control groups (39/41, 95.1% vs. 117/123, 95.1%; *P* > 0.999). During anti-TB treatment, paradoxical response occurred more frequently in the case group than in the control group (8/41, 19.5% vs. 4/123, 3.3%; *P* = 0.002).

**Table 2:**
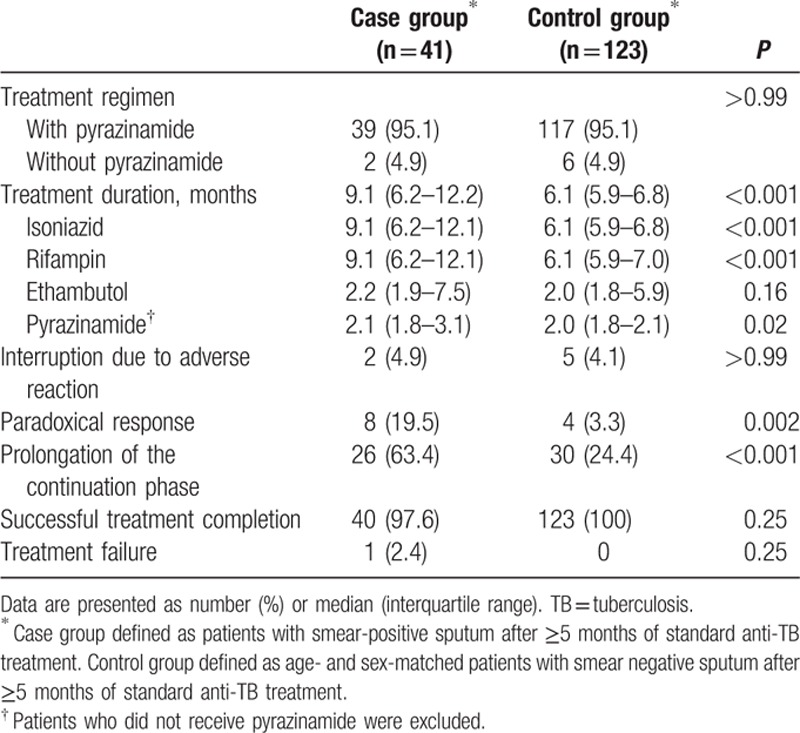
Treatment and outcomes according to sputum smear positivity after ≥5 months of anti-TB treatment.

Prolongation of the continuation phase of treatment more frequently occurred in the case group than in the control group (26/41, 63.4% vs. 30/123, 24.4%; *P* < 0.001). Total duration of treatment was longer in the case group than in the control group (9.1 months [IQR, 6.2–12.2 months] vs. 6.1 months [IQR, 5.9–6.8 months], *P* < 0.001). Treatment was successfully completed in all patients in the control group and in 40 patients (97.6%) in the case group without changes to second-line anti-TB treatment. There was no recurrence within 2 years after treatment completion in these patients.

Among the 41 patients in the case group, both clinical symptoms and radiographic lesions had improved at the time of positive smear after ≥5 months of treatment in all patients except one. One patient had a positive sputum culture after ≥5 months of treatment and was classified as treatment failure. Drug susceptibility tests revealed no acquired drug resistance. The addition of levofloxacin and prolongation of treatment duration resulted in sputum culture conversion in this patient.

### 3.4 Factors associated with positive sputum smear after ≥5 months of treatment

Candidate variables for multivariate logistic regression analysis included a history of previous treatment of pulmonary TB, cavitation, bilateral involvement, combined pleural effusion on chest radiography, and paradoxical response during anti-TB treatment, which were objective variables with *P* < 0.1 in univariate analysis (Table 3). In multivariate analysis, previous history of pulmonary TB treatment (adjusted odds ratio [aOR], 3.04; 95% confidence interval [CI], 1.09–8.49; *P* = 0.03), cavitation (aOR, 4.34; 95% CI, 1.86–10.11; *P* = 0.001), combined pleural effusion (aOR, 3.46; 95% CI, 1.15–10.42; *P* = 0.03) on chest radiography, and paradoxical response during anti-TB treatment (aOR, 7.24; 95% CI, 1.66–31.65; *P* = 0.02) were independently associated with positive sputum smear after ≥5 months of standard anti-TB treatment.

**Table 3:**
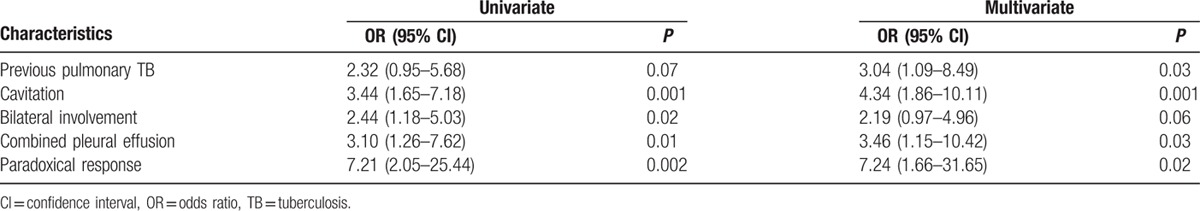
Factors associated with positive sputum smear after ≥5 months of treatment.

## 4 Discussion

In this study, among 1611 patients with culture-confirmed drug-susceptible pulmonary TB, 41 patients (2.5%) were smear-positive after ≥5 months of standard anti-TB treatment. Of these 41 patients, however, MTB was cultured in only 1 patient (2.4%). NTM were isolated in 7 (17.1%), and no viable mycobacteria were isolated in 33 (78.6%). The present study showed that smear positivity after ≥5 months of standard anti-TB treatment had no association with treatment failure and there was no need to change immediately to empirical second-line TB regimens in most patients. In addition, we found that a history of previous treatment of pulmonary TB, cavitation, bilateral involvement, combined pleural effusion on initial chest radiography, and paradoxical response during treatment were risk factors for smear-positivity after ≥5 months of standard anti-TB treatment in patients with drug-susceptible pulmonary TB.

Some previous studies evaluated the clinical significance of positive smears at 5 months or later during anti-TB treatment, although these studies included drug-resistant TB patients or did not have drug susceptibility results fully available.^[16–20,28]^ These studies reported that 2.2% to 9.0% of patients with pulmonary TB were smear-positive after ≥5 months of treatment.^[17–20,28]^ However, more than two-thirds of these patients were culture-negative, only 23% to 32% of these patients were culture-positive for mycobacteria, and the culture-positive rates for MTB, excluding NTM, were much lower in these patients.^[16–20,28]^ Our results were consistent with these data, although the culture-positive rate for MTB in our study (2.5%) was lower compared with previous studies, partly because all patients had documented drug-susceptible pulmonary TB.

An increase in the smear-positive, culture-negative phenomenon has been reported since the introduction of rifampin,^[29,30]^ presumably because the strong bactericidal action of rifampicin is on ribosomal components, leaving the cell wall stainable.^[19]^ Differentiation between dead bacilli and viable bacilli is often a significant challenge in clinical practice. Standard AFB smears and nucleic acid amplification tests could not distinguish live from dead bacilli.^[31]^ However, our study suggested that AFB sputum smear positivity with negative cultures after ≥5 months of treatment usually indicated dead bacilli and did not reflect treatment failure in patients with documented drug-susceptible pulmonary TB.

Patients who are classified with treatment failure receive a retreatment regimen including an injection drug or empiric second-line regimens for the presumptive diagnosis of multidrug-resistant TB.^[10,14,15]^ Correct identification of treatment failure is very important because retreatment regimens or empiric second-line regimens are inconvenient, costly, and potentially toxic for the patients. Therefore, assessment of treatment failure solely based on AFB smear status after ≥5 months of treatment may result in inappropriate medical decisions for many patients.^[14,15]^

We found that extensive tuberculosis lesions, such as cavitation, bilateral involvement, and combined pleural effusion on the chest radiography, were independent risk factors for positive smear results after ≥5 months of standard anti-TB treatment. Previous studies suggested that the incidence of unviable bacilli was higher in patients with extensive tuberculosis lesions, and the vastly increased bacillary load results in such an overwhelming number of dead bacilli that more time is required to clear the large load.^[18,30]^

The isolation of NTM during anti-TB treatment in patients with pulmonary TB is not uncommon (2.3%–14.2%).^[32–36]^ Patients with preexisting lung damage, such as previous pulmonary TB, are susceptible to NTM infection.^[37]^ In previous studies, TB patients with NTM isolation were more likely to have a history of previous treatment of pulmonary TB and advanced disease with cavitation.^[32,33]^ In our study, 17.1% (7/41) of patients had positive cultures for NTM in smear-positive sputum after ≥5 months of anti-TB treatment, and 31.7% (13/41) of patients had positive cultures for NTM in sputum, including subsequent sputum specimens. These findings suggest that a history of previous treatment of pulmonary TB and extensive pulmonary lesions could be associated with smear positivity due to NTM during anti-TB treatment. AFB smears cannot differentiate between MTB and NTM, and many NTM species are resistant to first-line anti-TB drugs in drug susceptibility tests.^[37,38]^ Therefore, NTM infection could be misdiagnosed as presumptive multidrug-resistant TB, especially in resource-limited settings.^[39–44]^

In this study, paradoxical response was also a risk factor for positive smear after ≥5 months of standard anti-TB treatment in patients with drug-susceptible pulmonary TB. Although the pathogenesis of paradoxical response remains unclear, an immune-rebound phenomenon may be occurring during anti-TB treatment, possibly enhanced by the release of MTB antigens during the destruction of infected macrophages.^[45]^ Therefore, differentiation between deterioration resulting from paradoxical response and treatment failure is important for appropriate management of these patients.^[46–48]^

In our study, the total duration of treatment was longer in patients with smear-positive sputum after ≥5 months of treatment than in control patients. When sputum smear was positive near the end of the scheduled treatment course in patients with drug-susceptible pulmonary TB, attending physicians tended to lengthen the continuation phase of treatment and await culture results and follow-up sputum examinations rather than changing the empirical second-line regimens.^[28]^ In our study, all patients except one showed clinical and radiographic improvement at the time of positive smear after ≥5 months of treatment. They continued standard anti-TB treatment and completed treatment successfully. This suggests that treatment failure should not be assessed based on sputum smear results only, and treatment outcomes should be carefully evaluated using clinical and radiographic responses. Prolongation of the continuation phase of treatment may be a reasonable strategy for these patients until confirmation of culture results.

There are several limitations to the present study. First, this study was conducted as a retrospective design in a single center. Results could vary widely between institutions and countries. Anti-TB drugs were self-administered with the support of trained nurses via outpatient therapy during the study period. Because adherence to treatment was not evaluated in this retrospective study, the study results cannot be generalized based on an assumption of good adherence. Second, a significant proportion of our patients could have been taking other medications because more than one-quarter of the study population was older than 65 years. However, possible drug–drug interactions, which could influence the effectiveness of anti-TB treatment, were not evaluated in the present study. Third, the viability of MTB in sputum cannot be precisely ascertained under routine standard culture conditions, even using both solid and liquid culture systems.^[49,50]^ There could be undetectable viable MTB present under conditions with resuscitation-promoting factors.^[50]^ Fourth, we did not evaluate the clinical usefulness of rapid molecular drug susceptibility testing. Recent guidelines recommend that patients who remain sputum smear-positive at completion of 3 months of treatment and patients in whom treatment has failed should be assessed for drug resistance using rapid molecular drug susceptibility testing (line probe assays or Xpert MTB/RIF) or conventional drug susceptibility testing.^[7,8]^ This approach could be applied to patients with smear-positive sputum after ≥5 months of treatment before declaring their cases as treatment failure.

In conclusion, smear-positive sputum after ≥5 months of standard anti-TB treatment was mainly because of nonviable MTB bacilli or NTM in patients with drug-susceptible pulmonary TB. AFB smear alone should not be used to assess treatment failure and careful examination of microbiologic status, including culture and drug susceptibility testing, is needed before making changes to a retreatment regimen or empirical second-line anti-TB regimen in these patients.
